# Outcomes of community-based suicide prevention program in primary health care of Iran

**DOI:** 10.1186/s13033-021-00492-w

**Published:** 2021-08-04

**Authors:** Hosein Azizi, Ali Fakhari, Mostafa Farahbakhsh, Elham Davtalab Esmaeili, Mohammad Mirzapour

**Affiliations:** 1 Social Determinants of Health Research Center, Health Management and Safety Promotion Research Institute, Tabriz University of Medical Sciences, Tabriz, Iran; 2 Research Center of Psychiatry and Behavioral Sciences, Tabriz University of Medical Sciences, Tabriz, Iran; 3 Department of Epidemiology and Biostatistics, School of Public Health, Tehran University of Medical Sciences, Tehran, Iran; 4 Medical Education Research Center, Tabriz University of Medical Sciences, Tabriz, Iran; 5 Road Traffic Injury Research Center, Tabriz University of Medical Sciences, Tabriz, Iran; 6 Malekan Health Center, Tabriz University of Medical Sciences, Tabriz, Iran

**Keywords:** Suicide prevention program, Primary health care, Re-attempt, Iran, Suicide, Self-injurious behavior

## Abstract

**Background:**

Suicidal management and prevention in communities, especially in its first stages, is an effective intervention for the health systems. However, in numerous societies most cases go undetected. Primary Health Care (PHC) is an effective place for the management of Suicide Prevention Programs (SPP). In Malekan County, a health community assessment found suicide as the most important health problem. A regional SPP was performed for suicide prevention during 2014–2017.

**Methods:**

This study was carried out in six steps: (1) Establishing a research team, (2) Improving a registry for suicidal behaviors (SBs), (3) Identifying local determinants of SBs, (4) Training healthcare providers, (5) Follow-up and monitoring of SBs, and (6) Public awareness campaigns. Our ultimate goal was to lower the rates of suicide, and suicide attempt (SA) by 15 and 20 %, respectively. Multiple logistic regression was used to estimate the adjusted odds ratios and the 95% confidence intervals.

**Results:**

A total of 821 SAs and 32 suicides were identified. The gender distribution for suicides was 70% males whereas SAs were 64% among females. The majority of suicides occurred in spring 18 (56.25%) while summer was the most common season among SAs 288 (35.8%). Almost 62 and 75% of suicides and SAs have used hanging and poisoning methods, respectively. Hanging increased suicide risk significantly (OR: 8.5, 95% CI 2.9–76.99). During the study, 93 life-skill and parenting education sessions were held. The incidence rates of suicide and SA decreased from 11.22, and 203 per 100,000 in 2013 to 2.63, and 157 in 2017, respectively. Similarly, the re-attempt to SAs ratio decreased from 12% to 2013 to 6.7% in 2017. Moreover, more than 8% of SBs were collected from adjacent Counties.

**Conclusions:**

At the study end, suicide, SA, and re-attempt were lowered by 75%, 22%, and 42%, respectively. The practical framework that achieved in this study could be used as a basis for developing future SPPs and suicide researches in the Iranian context. Furthermore, the various socio-economic and socio-cultural challenges highlight the need to consider a wide range of contextual factors when developing an SPP.

## Background

Suicidal management and prevention in communities, especially in its first stages, is an important intervention for health care system. Yet, in many societies most cases go undetected [1]. The degree of underreporting varied (overall: 10–30%) across countries [2]. In Norway and Finland, almost 10% of suicide cases could have been misclassified. However, in Malaysia and Egypt, the actual suicide cases could be more than 100% higher than the official suicide statistics [2, 3]. It is estimated that every year more than 1 million people die by suicide in the world, but these numbers are the tip of the iceberg and it is under-reported due to the lack of an effective Suicide Prevention Program (SPP), cultural stigma and the absence of registry for suicide [4, 5]. Despite these limitations, suicide is the second leading cause of mortality among people aged 15–29 years [6]. Suicide is the tenth cause of death in the United States where annually more than 35,000 deaths are attributed to suicide [7].

However, suicide and suicidal behaviors (SBs), are markedly challenging to be reliably tracked in health care and surveillance systems. The difficulty in documenting suicide has contributed to the lack of initiation and sustainment of SPP globally [5, 8]. Global suicide death reporting systems require significant improvement to provide valid and reliable data to design and implement programs [9, 10]. Since 2013, the World Health Organization (WHO) has had a global mental health action plan for reducing the rate of suicide by 10% in the world by 2020, but merely 18% of countries have a registry for suicide.

Primary Health Care (PHC) system is an effective and appropriate place for developing and management of SPPs. PHC services are the most readily available means of health care which are accessible and with high rates of regular contact so that many health service providers in primary care can play a noticeable role in suicide prevention [11].

In Iran, suicide is considered the fifth cause of death [12]. The incidence rate of suicide has increased over the past decades, especially in East Azarbaijan Province where there are limited studies on the subject [13]. Iran has a suicide rate between 1.4 and 29.6 per 100,000 population [14].

According to the report of the WHO, SPP is not only critical for persons and families but moreover it hugely profits the health care system and welfare of communities and society [15]. A community health assessment indicated that SB is the most important health problem in Malekan County, East Azarbaijan. A regional SPP was developed and implemented for suicide and SB prevention in the PHC setting during the period 2014–2017 years [13, 16]. The findings and implications of this interventional study can be useful for health care systems. Therefore, the main objective of this paper is to outline the findings and outcomes of this interventional program in the reduction of suicide and SB.

## Methods

### Study design and setting

**Fig. 1:**
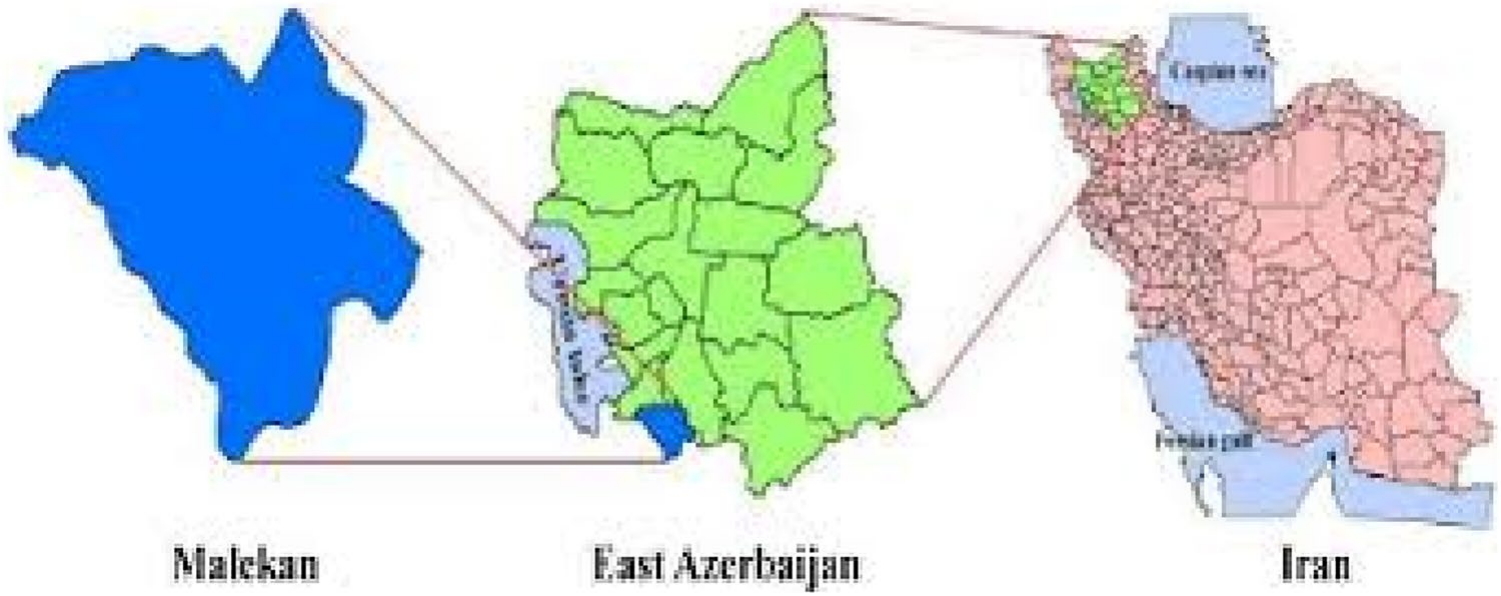
Location of Malekan County in Iran

A regional community-based SPP was performed for suicide and SB prevention in PHC system of Malekan County, East Azarbaijan Province, Iran during 2014–2017. Malekan County is located in the northwest of Iran with 117,000 population in 2018 (Fig. 1). Native language of all its population is Azeri and all of them are Muslims. Almost, 70% of the County population live in rural areas. Their main occupations are farming or farming-related.

Community health needs assessment by the health network of Malekan County revealed that suicide and SBs are among the most important public health concerns. This community health assessment was performed in 8 steps based on North Carolina community assessment model and included (1) Establishing a Community Assessment Team (2) Collecting Community Data (3) Analyzing the County Health Data Book (4) Combining the County’s Health Statistics with Community Data (5) Reporting to the Community (6) Selecting Health Priorities (7) Creating the Community Assessment Document (8) Developing a Community Health Action Plan [17]. Consequently, we performed a community-based SPP as stated in the eighth step (implementing an action plan for suicide prevention). This project was conducted in collaboration with the Department of Mental Health in Malekan County Health Network and the Research Center of Psychiatry and Behavior Sciences at Tabriz University of Medical Sciences. The target and study population were the general population of Malekan County, especially hotspot areas. In Iran, medical universities are responsible for the health system in each province. Iranian health system is based on Health Care Networks and thus the PHC provides the first line of health care services to assure access of all urban and rural populations by family physicians and various types of Health Service Providers (HSP). When patients need special health services, they are referred to the second level (county hospitals) by family physicians, and when higher specialty services are required, patients are referred to province referral hospitals with high specialty services [18, 19].

## Procedure

Six steps were carried out for developing and implementing interventions including.

### Establishing an action research team

This study was a process. For this process to be meaningful, team members and the health service providers from throughout the health system must be mobilized and all be involved during the research. We included academic members (Psychiatrists and Psychologists) from the Department of Psychiatry (School of Medicine) and executives from the Department of Mental Health (the provincial Deputy of Health), Tabriz University of Medical Sciences, and officers and health service providers from the County Health Network. The health service providers played as the engineer force in this project. Moreover, this team was used to select and prioritize effective programs and interventions for developing and implementing SPP.

### Developing and improving a registry for suicidal behaviors

The national registry for suicide was launched in 2009 in Iran and was based on reports from medical universities. In the counties, there isn’t an electronic suicide registry system. The suicides are reported to province medical universities based on paper formats. Malekan County isn’t so large and as it is known, suicides are under-reported especially in small communities because of cultural barriers, stigma, and lack of an effective surveillance system.

Accordingly, we performed several efforts to increase the coverage of suicide registry in the county including (1) developing and using a simple and rapid checklist for collecting primary data in the emergency room of the hospitals, (2) Collecting information of SBs who were referred to the adjacent counties including Miyandowab, Bonab, and Maragheh, (3) Using native Community Health Workers (CHWs) (*Behvarz* in Persian) to obtain valid information from SBs and improving coverage of suicide and SA reporting and registration, (4) Improving intra-sectoral collaborations between Deputy of Health and Deputy of Treatment, (5) Improving inter-sectoral collaboration with clergy, village administrators and council members, municipalities, and governors with several advocacy activities.

### Identifying local determinants of SBs

Suicide and SBs are affected strongly by social and cultural aspects. To identify and a better understanding of local effective factors of SBs, we carried out semi-structured and face-to-face interviews with most SBs by trained clinical psychologists. The interviews were performed with the most nearby individuals of family members including parents, spouse or siblings instead of the suicide (died) cases. A valid and comprehensive questionnaire was used to collect effective factors on suicide and SBs such as demographic characteristics, socio-economic status, history of SA, psychological and depressive disorders, applied methods for suicide, season, and details of SBs, cultural and religious beliefs, and psychiatry and other determinants of suicide in a single and private setting.

The validity of this questionnaire was determined based on expert opinions including two psychiatrists, one epidemiologist, one community mental health expert, and one psychologist. Then some questions were revised. Its reliability was assessed with Cronbach’s alpha test (α = 0.78) among 20 participants.

### Training healthcare providers (gatekeepers)

CHWs in PHC system of Iran have face-to-face contact with large numbers of community members as part of their usual routine performance. Each CHW provides mainly preventive healthcare services to approximately 1000 people. CHWs are supported and supervised by Community Health Extension Workers (CHEW) who are trained health care workers, psychologists, or public health staff, based on the linked health facilities. Many workshops and training sessions were conducted for CHWs, CHEWs, family Physicians, psychologists, and public health staff about details of the program and their practice. A comprehensive instruction included program details that were made available to all healthcare providers.

### Follow-up and monitoring of SBs to prevent re-attempts (risk assessment for suicide and depression)

**Fig. 2:**
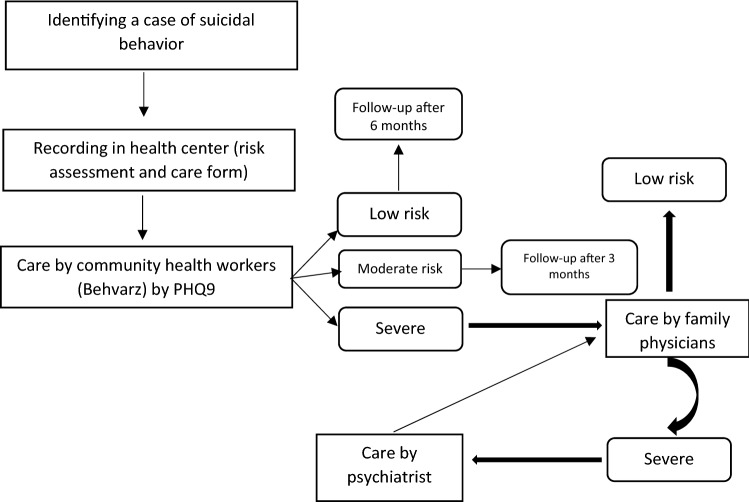
Follow-up monitoring of suicidal behaviors to prevent re-attempts

History of SB and depressive disorders are robust predictors for suicide and future attempts. Case management of SAs was one of the most effective interventions to prevent re-attempts in the future. Figure 2 shows the algorithm of the follow up and monitoring of the SBs to prevent re-attempts.

After each SB has occurred, a paper-based file was created in the nearest health center. It was included the socio-demographic status of SBs and risk factors, medical histories, as well as depression and suicide risk assessment via Item 9 of the Patient Health Questionnaire (PHQ9). PHQ evaluates passive thoughts of death or self-injury within the last two weeks, and is often used to screen depressed patients for suicide risk [20].

All SB cases were followed by CHWs. According to the expert panel of this study, the risk of suicide and depression was assessed by PHQ. This tool was scored on a 3-point scale. The minimal (low-risk) score in PHQ ranges from 0 to 4, moderate risk scores range from 5 to 14, and severe risk is considered ≥ 15 [21].

Moderate and low-risk cases were followed up (re-assessed) by CHWs after 3 and 6 months, respectively. Severe cases were referred to the family physicians. Severe cases, after receiving care of family physicians, were referred to the psychiatrist in the County hospital (Farabi Hospital) if they had needed more specialized services or identified any psychiatric disorder. To prevent re-attempt cases, psychiatrists were used structured interviews to diagnose and treatment of depressive and other psychological disorders.

### Public awareness campaigns in hotspots

In hot-spot areas, we held life-skills conferences and educating sessions with inter-sectoral collaborations (stakeholders and supporters of villages and cities) for heads of households, women, teenagers and youth and other at-risk populations. The life-skills education sessions were held by clinical psychologists and included parenting, effective communication, problem-solving, decision making, creative and critical thinking, and coping with stressful life events. These workshops were held in public sites such as the health center and mosques to be accessible to the general public.

Comprehensive and community-based campaigns have been implemented for improving knowledge, awareness, and attitude as well as depressive disorders and mental health-related issues of large numbers of the community. Coverage of free access to the mental health services included providing free drugs for major psychiatric disorders, school-based training and health-related services, marital counseling and other services were increased. Educational packages, posters, pamphlets, and banners were produced and distributed throughout the city and villages. The social stigma due to mental disorders and receiving psychological and mental health services from the public sector was reduced with performing the various training and mobilization practices.

### Outcomes

Primary outcome was considered reduction of completed suicide. Secondary outcome was reduction of rates of SA and re-attempt. Our ultimate goal was to lower the rates of suicide, SA, and re-attempt till the study end by 15%, 20% and 30%, respectively.

### Statistical analysis

The SPSS software (version 19.0, Chicago, IL, USA) was used for data analysis. Descriptive statistics and graphs were used to describe frequencies and ratios. Data normality was checked using the Kolmogorov-Smirnov test. Chi-square (χ2) test was used to compare binary or more categorical variables. T-test was used for comparison of continuous variables. Simple and multiple logistic regressions were used to estimate the crude and adjusted odds ratios (ORs) and 95% confidence intervals (CIs) for suicide risk. In all tests, the confidence interval was considered 95% and P-value < 0.05 was significant.

## Results

Table 1 summarizes suggested interventions for suicide and SB prevention in the general population in the Malekan County. Interventions for suicide prevention included follow-up monitoring of attempters, suicide risk assessment and treatment of depressive disorders, improving SBs registration, identifying risk factors of SBs, and implementation of public education campaigns in hotspots (Table 2).

**Table 1:** Implemented interventions for suicide and suicide attempt preventions in Malekan County during 2014–17

Order	Interventions
1	Establishing an action research team
2	Improving registry for suicidal behaviors
3	Follow-up monitoring of suicide attempters to prevent re-attempt (depression and suicide risk assessment)
4	Training healthcare providers (Gatekeeper)
5	Identifying local determinants of suicidal behaviors and risk factors
6	Public awareness campaigns in hotspots

**Table 2:** Socio-demographic characteristics and risk of suicide between suicides and suicide attempters in the Malekan County, from 2014–2017

Variables	Attempters (N = 821)	Suicides (N = 32)	OR (95% CI)	P-value
Gender				
Female	525 (63.95)	9 (28.12)	1	1
Male	296 (36.05)	23 (71.9)	4.6 (1.99–10.63)	0.001
Age				
10–25	504 (61.39)	9 (28.125)	1	1
26–40	241 (29.35)	18 (56.25)	4.22 (1.75–10.15)	0.001
≥ 40	76 (9.25)	5 (15.63)	3.76 (1.1–12.92)	0.035
Occupation				
Student	181 (22.05)	7 (21.87)	1	1
Farming related	24 (2.92)	2 (6.25)	2.42 (0.36–16.03)	0.357
Housewife	540 (66.77)	5 (15.63)	0.23 (0.07–0.80)	0.021
Unemployed or free	76 (9.25)	18 (56.25)	1.43 (0.68–3.17)	0.038
Marital status				
Single	140 (17.05)	10 (31.25)	1	1
Married	611 (74.42)	21 (65.63)	0.47 (0.20–1.12)	0.091
Widow and Divorced	70 (8.53)	1 (3.12)	0.2 (0.23–1.73)	0.144
Educational level				
Primary school	277 (33.74)	10 (31.25)	1	1
Secondary school	427 (52.00)	19 (59.38)	1.23 (0.53–4.21)	0.623
High school and Academic	118 (14.37)	3 (9.37)	0.70 (0.17–2.82)	0.626
Family size				
2 ≥	155 (18.83)	4 (12.5)	1.37 (0.72–3.27)	0.317
3–4	443 (53.9)	16 (50.00)		
≥ 4	223 (27.16)	12 (37.5)		
Income (Rials)				
< 5 million	383 (46.65)	8 (25.00)	1	1
5 –10 million	304 (37.02)	13 (40.62)	2.05 (0.79–5.3)	0.138
10–20 million	91 (11.08)	4 (12.5)	2.11 (0.56–7.88)	0.263
> 20 million	43 (5.24)	7 (21.88)	7.87 (2.24–27.57)	0.001
Resident				
Urban	166 (20.22)	2 (6.25)	1	1
Rural	655 (79.78)	30 (93.75)	3.22 (0.812–12.78)	0.079
Live alone	43 (5.23)	1 (3.12)	0.94 (0.91- 1.19)	0.530
Substance abuse	45 (5.48)	2 (6.250	1.11 (1.00–1.27)	0.042
Alcohol Consumption (daily)	41 (4.99)	11 (34.37)	1.43 (1.12–1.85)	0.001
Smoking (daily)	147 (17.90)	3 (9.37)	1.49 (1.12- 2.06)	0.001

Table 2 indicates the socio-demographic status of suicide cases and attempters in Malekan County during 2014–2017. A total of 821 SA and 32 suicide cases were found during the study course. The gender distribution for suicides was 70% male while the majority (64%) of attempters were females. A significant association was found between completed suicide and male gender, age, occupation, family income, smoking, alcohol, and substance abuse (P ≤ 0.05). Regarding occupation, suicide deaths were lower among housewives (P = 0.021). Moreover, higher education decreased the chance of completed suicide while its association with suicide was insignificant (OR: 0.70, 95% CI 0.17–2.82).

Table 3 shows the descriptive epidemiology of participants in the County from 2014 to 2017. The hanging method (62%) was the most frequent among suicide cases while the poisoning (75%) was the most prevalent among SAs. The hanging method markedly increased suicide risk, 8.5 times (OR: 8.5, 95% CI 2.9–76.99). Moreover, majority (56.25%) of suicides occurred in spring.

**Table 3:** Descriptive epidemiology (methods, time and place) of suicide and suicide attempts in Malekan County during 2014–2017

Variable	Suicide N = 32	Attempters N = 821	Crude OR (95% CI)	P-value
Methods				
Hanging	20 (62.5)	11 (1.34)	8.5 (2.9–76.99)	0.001
Poisoning	8 (25)	613 (74.66)	0.63 (0.18–2.24)	0.483
Self-injury	2 (6.25)	159 (19.37)	0.446 (0.12–1.78)	0.236
Self-burning	2 (6.25)	38 (4.63)	1	1
History of attempt				
Yes	8 (25.0)	47 (5.72)	2.59 (1.086–6.17)	0.028
No	24 (75.0)	774 (94.28)	1	1
Place				
Residential building	26 (81.25)	747 (90.98)	2.23 (1.18–4.21)	0.013
Un-residential building	1 (3.125)	21 (2.55)		
Outside	5 (15.62)	53 (6.45)		
Seasonal				
Spring	18 (56.25)	150 (18.27)	1	1
Summer	3 (9.37)	288 (35.8)	0.089 (0.024–0.32)	0.001
Autumn	2 (6.25)	259 (31.54)	0.066 (0.014–0.30)	0.001
Winter	9 (28.12)	24 (2.92)	2.21 (0.70–6.96)	0.174

More than one-third (1/3) of suicides had at least one time the history of SA. So, it was significantly associated with suicide risk (OR: 2.59, 95% CI 1.086–6.17). Likewise, majority of suicides have occurred in the residential building (81.25%).

Table 4 shows the results of multiple logistic regression analysis and estimating Adjusted Odds Ratios (AORs) and 95 % Confidence Intervals (CIs) for suicide risk. After adjusting for the potential confounders, it was found age (26–40), male gender, higher income (more than 10 million Rials), and unemployment to be associated with the risk of suicide. Likewise, history of SA (AOR = 2.23, 95% CI 1.70–6.45), hanging method (AOR = 12.62, 95% CI 3.14–28.02), and season (spring) (AOR = 3.56, 95 % CI 2.19–9.63) were increased suicide risk.

**Table 4:** Measure of association and 95% confidence intervals for suicide risk and effective factors by multiple logistic regression

Variables	Adjusted OR (95% CI)	P-value
Age		
10–25	1	1
26–40	6.34 (2.1–19.15)	0.001
≥ 40	4.92 (0.8–30.58)	0.088
Gender		
Female	1	1
Male	3.48 (1.32–9.24)	0.012
Income (Rials)		
≤ 10 million	1	1
> 10 million	2.68 (1.06–5.41)	0.049
Occupation		
Student	1	1
Farming or farming related	2.49 (0.138–45.05)	0.535
Housewife	0.198 (0.045–0.86)	0.032
Self-employed or unemployment	6.88 (1.73–27.53)	0.006
Attempt method		
Hanging	12.62 (3.14–28.02)	0.001
History of SA		
Yes	2.23 (1.70–6.45)	0.002
No	1	1
Season		
Spring	3.56 (2.19–9.63)	0.001

Among all 821 SA which were assessed by PHQ 9 inventory tool, more than 288 (35 %) of SAs had a high risk (severe) for suicide (Table 5). During the implementation phase, a total of 93 life-skill and parenting education sessions were presented for targeted groups of adolescences, parents, and household heads in the hotspot areas of the County (Table 6).

**Table 5:** Suicide risk assessment among attempters by PHQ9

Year	Attempters	PHQ scores		
		Low (0–4)	Moderate (5–14)	Severe (15≤)
2014	251	51 (20.32)	114 (45.42)	86 (34.26)
2015	202	42 (2079)	83 (41.09)	77 (38.11)
2016	189	45 (23.8)	80 (42.32)	64 (33.86)
2017	179	43 (24.02)	75 (41.90)	61 (34.08)
Total	821	181 (22.05)	352 (42.87)	288 (35.08)

**Table 6:** Life-skills education sessions in the hot-spots during the suicide prevention program

Region/Area	Type of educating sessions	Number of sessions*	Target group
Rural	Life-skill*	8	Adolescents, young people and Households heads
	Parenting	38	Parents with children 12–2 years old
	Total	46	
Urban	Life-skill*	15	Adolescents, young people and Households heads
	Parenting	32	Parents with children 12 –2 years old
	Total	47	
Total		93	

Each session performed at least 20 participants and lasted for 45 min

* With component of effective communication, problem-solving, decision making, creative and critical thinking, and coping with stressful life events

Table 7 indicates the effect of the interventions on primary (suicide) and secondary outcomes (attempt and re-attempt) in Malekan County during 2014-17. Interventions demonstrated that both suicide and SA rates had declining trends in the County so that at the end of the study, 75 and 22 % of completed suicide and SA decreased compared to before the study (2013). The incidence rate of suicide decreased in County from 11.22/100,000 in 2013 (before the study) to 2.63 in 2017. Likewise, SA rate decreased from 203/100,000 in 2013 to 157 in 2017. Moreover, out of 821 suicide attempters, 67 (8.16 %) were collected from adjacent Counties included Miyandowab, Bonab and Marageh.

**Table 7:** Primary and secondary outcomes^*^ of suicide prevention program in Malekan County, during 2014–2017

Year	Population	Suicide (N = 32)		Suicide attempters		Attempt to suicide ratio
		N	Rate (per 100,000)	Attempters N = 821	Collected through adjacent counties ^**^	
2013 (before study)	107,000	12	11.22	217 (203 per 100,000)	No data available	18.083
2014	109,000	9	8.25	251 (230 per 100,000)	17 (6.77)	27.88
2015	111,000	5	4.50	202 (182 per 100,000)	21 (10.4)	40.40
2016	112,000	5	4.46	189 (169 per 100,000)	16 (8.4)	37.50
2017	114,000	3	2.63	179 (157 per 100,000)	13 (7.25)	59.66
Total		32	Reduction at the end = 75% (11.22–2.63/11.22)	Reduction at the end = 22% (203 − 157 /203)	67 (8.16)	-

*The primary outcome: suicide; Secondary outcome: suicide attempt and re-attempt

**Miyandowab, Bonab and Maragheh

**Fig. 3:**
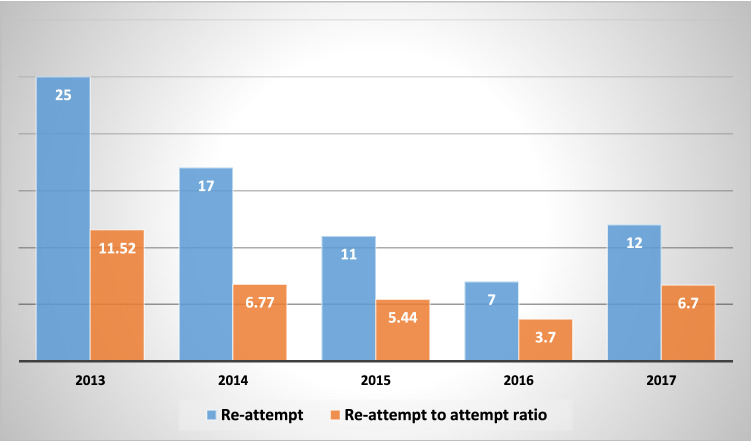
Trend of re-attempt distribution and ratio of re-attempt to attempt during study period (2014–2017) in Malekan County, Iran

Figure 3 shows the implications of SPP on re-attempt trends and proportions. A decreasing trend was found during the study period from 2014 to 2017. Re-attempt to attempt ratio was reduced from almost 12% in 2013 (before the study) to 6.7% in 2017. Compared to before the study, the re-attempt measure decreased 42%.

## Discussion

The present community-based SPP was implemented in Malekan County during 2014–2017. Primary outcome aimed to lower by 15% of completed suicide, and secondary outcomes also 20 and 30% in SA and re-attempts, respectively. We found that interventions markedly decreased the rates of suicide and SA in the general population of Malekan County in all years of the study period. Suicide and SA were decreased from 12 to 203 per 100,000 in 2013 to 2.63 and 157 in 2017, respectively. In other words, at the end of this intervention study, almost 75 and 22% of suicide and suicide attempts were decreased, respectively.

Moreover, at the study end, a 42% decrease in re-attempt proportion indicates the effectiveness of the study interventions. In the first year of the study (2014), developing a community-based registry for SB and collecting SBs from adjunct counties increased the number of SAs. Despite this, the trend of suicide, SA, and re-attempts decreased during the study period with a smooth slope.

Keeping up with the literature and the WHO report for suicide prevention at the communities, developing and improving community-based registry for SB, case management of persons who attempted suicide, identification and treatment of depression, training and public awareness campaigns and conducting research were found the most important and effective interventions to achieve the present study outcomes [22–26].

In our opinion, this community-based prevention and surveillance program is an effective and most applied health system research. We have tried to resolve this problem with the assistance and support of the health system after identifying the problem, similar to action research. We organized the health system in line with our purpose for achieving the study outcome measures and improving a problem-solving perspective between healthcare providers. It was needed more practice and coordination.

We tried to obtain the support of health managers, county decision-makers and stakeholders with many advocacies efforts. We have used the influence of the clergy, and stockholders among County people for implementing the study interventions and educational programs. The evidence has shown effective efforts to reduce suicide would need pooled interventions by different providers in several fields [27].

Furthermore, in Iran, health system and health services are provided based on PHC and Health Care Networks. PHC is an effective and appropriate place for developing and management of SPPs with various types of healthcare providers in particular community health workers. Community healthcare workers have face-to-face contact with large numbers of community members as part of their routine performance. Around the world, many suicide prevention programs in various designs (RCT, descriptive, qualitative and other methods) had been performed [28]. However, this study carried out in limited resource with community-based interventions, as could provide a comprehensive framework for low and middle-income settings.

In most studies, SPP and strategies focus on increasing access to psychological health services for at-risk people via general practitioners and restricting access to means of self-harm/suicide [29–31]. In addition to the above-mentioned interventions, this study emphasized remarkable interventions that included case registration, case management of attempters, public awareness campaigns, treatment of depression, training of health service providers, conducting research, and social and economic factors related to suicide prevention.

The annual decrease of re-attempters shows the appropriateness and effectiveness of the present study interventions. Management of persons who attempted suicide is one of the most effective recommendations also reported by the WHO. The risk of re-attempt after SA was high in the months following an attempt. This finding is also in agreement with previous studies [32, 33].

Globally suicide is a priority condition. A national SPP is necessary through a stepwise approach at the local level. A SPP at the national level not only explains the aim and magnitude of the problem but more remarkably, identifies that SB is a critical public health problem. SPP should recommend a comprehensive evaluation and monitoring framework to measure the effectiveness of interventions. Prevention of suicide is a collective responsibility and should be organized by the political commitment of governments and civil society throughout the world [34].

Moreover, findings of the present study showed that demographic characteristics (male gender, age 26–40, living in rural area), and socio-economic status (low-education, low-occupational status (unemployment), high-income), and behavioral-factors (tobacco and alcohol abuse) were associated with completed suicide. In agreement with our findings, a systematic review study revealed that low socio-economic status, low-education, and low occupational status were related to suicide risk [35]. Likewise, a study from England and other countries found that socio-demographic characteristics and low socio-economic status were directly associated with suicide and SBs [36, 37]. In the present study, suicide was common among high-income families while SAs were prevalent among low-income families. Another review study found low economic status, and unemployment to be associated with SBs [38].

### Strengths and limitations

The main strength of this study was the use of multidimensional practical interventions that resulted in significant achievement on suicide prevention. Another strength was using and integrating action and research to achieve study measures.

The present study had a limitation. This study was conducted in a small area with limited population. So, application of its methods and findings in large communities should be with caution.

## Conclusions

Findings at the study end indicated that interventions achieved predetermined aims and outcomes. Primary outcome (suicide) lowered by 75%, and secondary outcomes (attempt and re-attempt) reduced 22 and 42%, respectively.

In this study, developing and improving a registry for suicide and suicidal behaviors, case management of suicide attempters, training healthcare providers, public awareness campaigns in hot spots, and conducting local-research were found as effective interventions and strategies for suicide prevention. The findings of this study can provide valuable evidence and framework for the Malekan County and national mental health programs.

Moreover, findings showed that socio-economic status and socio-demographic characteristics were associated with lethal suicide. These economic, structural, and socio-cultural issues serve as barriers to the implementation of SPPs. These findings challenge the personal risk-factor models of SPP and highlight the need to consider a wide range of contextual and socio-economic, and socio-cultural factors when implementing SPPs [39].

The practical framework that appeared in this study could be used to develop SPPs and suicide researches in Iranian context. This study also provides an excellent prospect to bridge the gap between research and field programs and/or policy. These all recommend the need to reinforce the awareness about SBs and evaluate the efficiency of the national health approach in addressing the issues of suicide and SBs [40].

### Specific suggestions

Improving suicide registry coverage: this finding suggests that the first pillar for developing an effective SPP and/or strategy is to establish an electronic registry for suicide which is also emphasized by the WHO.

Advocacy and inter-sectoral collaborations: Strong political commitment and significant social actions are needed at the grass-root level for implementing an SPP. Therefore, the SPP should be customized with a comprehensive policy and with a multidimensional approach that includes active cooperation between the various sectors of government, health sectors, social welfare, stakeholders, clerics, urban and rural development experts, legal affairs, and especially general population and their participation [41].

Combined actions with local researches (action-research): Predictors and patterns of suicide and suicidal behaviors are affected strongly by local level religion and customs. Therefore, to achieve significant implications, should integrate research and action, and the interventions should be developed based on local-evidence. Despite the amplified focus of health managers and policymakers on the significance of suicide prevention strategies, findings confirm an evidence gap in research knowledge regarding program development, planning, and effective outcomes, and there remains a need for the integration of research and knowledge utilization [42, 43].

Case management of SBs: Findings of the present study revealed that integrated case management and follow-up monitoring of SBs are low-cost and feasible strategies that can reduce suicide and SBs. However, sub-interventions should be selected via high-evidence and expert testimony.

## Data Availability

The datasets generated and/or analyzed during the current study are not publicly available due to the sensitivity of the suicide issue and the confidentiality of the information, the consent of the sponsoring organization is required. But are available from the corresponding author on reasonable request.

## Abbreviations

SPP: Suicide prevention program
SBs: Suicidal behaviors
SA: Suicide attempt
PHC: Primary health care
CHWs: Community health workers
PHQ: Patient health questionnaire
